# The Influence of Mechanical Properties of Laser-Melted Tungsten Carbide Composite with Nickel/Cobalt Ingredients

**DOI:** 10.3390/ma17225636

**Published:** 2024-11-18

**Authors:** Xiao-Dong Wang, Ming-Der Jean

**Affiliations:** College of Arts and Design, Jimei University, Xiamen 361021, China; 200561000056@jmu.edu.cn

**Keywords:** Tungsten carbide, interaction model, analysis of variance, mechanical properties, metal matrix composite, laser cladding

## Abstract

This study used statistical tools to optimise WC/Co/Ni welds and model construction to improve the mechanical properties of coatings by laser cladding. The effect of the parameters on the wear performance of the weld was determined by analysis of variance. In addition, a polynomial model was constructed using the response surface method based on the experimental data of the orthogonal array designed by Taguchi. The experimental results show that there are white initial precipitation carbides and grey areas of WC mixed with Co and Ni compounds, while less wear and less plastic deformation are observed with WC/Co/Ni alloys. By adding Co/Ni alloys, the composite coating extension is seen to have good anti-wear performance. Based on the regression model, a pairwise interaction model was successfully constructed and further modelling of the 3D contour of the wear behaviour was explored. Comparing all the experiments, the predictions of the interaction model were found to be reliable, with an average error of 8.75%. The findings show that there is a very close match between the predicted values of RSM for wear performance and the experimental data, which proves the effectiveness of the Taguchi design-based RSM in improving the mechanical properties of laser cladding.

## 1. Introduction

The use of ceramic matrix composites in the form of coatings for hard surface environments with high temperature, wear, corrosion, impact and fatigue resistance has attracted much attention. This is particularly true for surface-engineered components for industrial applications [1,2,3]. Recently, much attention has been paid to the use of ceramic matrix composites (including carbides, nitrides and borides) on steel and non-ferrous alloys, which have excellent substrate reinforcement properties and have proven to be an excellent protective material in manufacturing [4,5]. Tungsten carbide (WC), especially sintered carbides, has many advantageous properties such as high hardness, some plasticity and good wettability compared with other high-temperature oxidation-resistant and wear-resistant carbides, such as SiC, TiC, TiB_2_ and Mo_2_Si [6,7,8]. Frequently used in applications such as cutting tools, turbine blades, automotive engine valves and other wear-resistant parts, these unique properties make them suitable for a wide range of applications due to their low cost, high pressure, abrasion and corrosion resistance [9].

Recently, WC has been used to modify the surface properties of components through techniques such as arc welding [10], thermal spraying [7], nitriding, sputtering [11] and laser cladding [12]. These techniques protect industrial components from wear [13], corrosion [14] and fatigue damage [15]. Compared with the other technologies above, laser cladding coating has the advantages of dense microstructure and strong metallurgical bonding with the substrate. It has promising potential applications in the manufacturing of metal–ceramic composite coatings by laser cladding. Laser cladding has been widely studied. It is an effective surface modification technology for improving the surface properties of metal matrix composites and their alloys. Due to their high melting point, hardness and brittleness, WC alloys are prone to porosity and cracking in the liquid state. They are also difficult to machine. As a result, some typical cemented carbide products, such as metal matrix composites, struggle to meet the requirements of industrial components in severe working media. In addition, a number of disadvantages of cemented carbides have been noted, including reduced strength, inhomogeneous distribution, concentration of thermal stresses and the formation of cracks, especially at high proportions of C in WC coatings, thus limiting the applications of WC.

These challenges have led to the investigation of powder-based techniques with ceramic metal composite coatings. Autorheological alloy powders are a series of alloys with low melting points and good self-shielding effects, which have been successfully applied to laser cladding, such as Ni-based alloys [16], Co-based alloys [17] and Fe-based alloys [18]. This is because they have good wetting and spreading properties. The opportunity to extend some of these useful alloys to high-cement WC is significant and could be further investigated to improve the detrimental properties of WC. In conclusion, there is an urgent necessity to better understand the effect of self-melting alloys, such as Co or Ni, Co/Ni, etc., mixed with WC in metal matrix composites on the mechanical properties of laser WC coatings [19].

The available literature discusses the manufacturing of metal–ceramic coatings by laser cladding using metal matrix composites coupled with self-fusing alloys. For example, the melt zone crack initiation and propagation behaviour of WC-reinforced Fe-based metal matrix composites was investigated by Farahmand et al. Cracks mostly extend along the very brittle eutectic phase, while the thin interface facilitates the transfer of stresses from the particles to the matrix [20]. Paul et al. investigated pulsed Nd:YAG laser cladding of mild steel with WC-12Co welds. The results show that the deposited WC-Co surfaces are completely dense and crack-free, with excellent metallurgical bonding and low dilution. No melting of WC particles in the Co matrix was observed [13]. Zhou et al. analysed a Ni-based WC composite coating using an elliptical spot laser induction hybrid fast fusion technique. This works up to four times more efficiently than typical laser cladding with crack-free ceramic-to-metal coatings [21,22]. The development of micron and nanoscale WC-12Co coatings on stainless steel by means of microwave hybrid coating technology was investigated by Zafar et al. The flexural strength of the nano-surfaced layer is approximately 14% more than that of the micron-surfaced layers [23]. Zhou et al. investigated the microstructure and wear properties of NiCrBSi/50 wt.% WC composite coatings using both laser cladding and laser induction hybrid welding [24]. Shu et al. conducted high volume fraction WC-enhanced Ni-based coatings synthesised in situ by laser cladding. This work has shown that multilayer coatings have the highest hardness of any coating, approximately 3.7 times the hardness of the substrate [25]. Zhou et al. found that WC composites with Ni-based coatings could be rapidly deposited with elliptical spots by means of laser-induced mixing [21]. Zhang et al. studied the effect of wear and electrochemical corrosion behaviour of Ni-Cu alloys on WC-12Co composite coatings by laser cladding [25]. In addition, the microstructure evolution of satellite 6 with different volume fractions of WC by laser cladding was investigated by Zhong et al. [8].

It is known from the above literature that composites are usually formed by adding transition metals such as Co-based or Ni-based alloys to brittle carbides as the bonding phase material to reduce the structural defects of highly brittle carbides during laser cladding [26,27,28]. Yet, the research on laser cladding of WC/Co/Ni composites on steel substrates is still limited in the literature [16,29,30]. However, laser cladding is a highly variable process with a lot of uncertainty and fuzzy knowledge, often requiring empirical decisions with unsatisfactory results [20,31,32]. To control the effect of mechanical properties of metal–ceramic composite deposits at multiple variables, it is very difficult to use the trial-and-error method [31,32]. Therefore, some new efforts are required [33,34,35]. Response surface methodology (RSM) is well suited to RPD problems and general process robustness studies, where it applies statistical and mathematical techniques [36,37,38]. It has great application in the design, development and manufacturing of new products [39,40,41]. Although several studies have been carried out on various WC coatings, no optimisation studies using the RSM-based Taguchi method are available for WC/Co/Ni coatings prepared by laser cladding [42,43,44]. Therefore, the application of RSM helps to study the effect of WC/Co/Ni coatings on mechanical properties that can be optimised and to obtain better results of mechanical properties for the coatings [45,46,47].

In this study, the WC-Ni/Co blend powders were deposited by laser melting that formed a metal–ceramic composite weld, thereby improving the performance of the high-hardness WC. In addition, a Taguchi test-based RSM was developed by identifying the appropriate combination of control factors that could improve the mechanical properties of laser-coated WC welds [48,49,50]. Furthermore, the residual stress behaviour, microstructural evolution, wear characteristics, hardness and fracture crack formation of WC deposits with different Co/Ni ratios in composite welds by laser cladding were studied [51,52,53,54]. The impact of the parameters on the response was evaluated. For a better understanding of the mechanical properties of laser-coated samples, the parameters were optimised.

## 2. Experimental Procedure

### Materials and Preparations

In this study, the fibre laser cladding system consists of an IPG YLS-3000 fibre laser, a 6-axis robot, an induction power supply, a computer numeric control system and a powder feeder (HUST III). The system uses a fibre laser with a maximum power of 3000 W and a wavelength of 1.07 μm. The percentages of W are 80%, 90% and 100%, respectively, for WC/Co/Ni blends. The base metal, measuring 40 × 20 × 10 mm^3^, is 45 grade and 40Cr steel. Table 1 shows the chemical composition of the Co and Ni-based alloys used for the WC welds. A scanning electron microscope (Hitachi S-2600H, Tokyo, Japan) was used to examine the microstructure of the WC coatings and the etching test was carried out using a Nital for 5 to 10 s. In the wear test, the weld surface was subjected to a reciprocating wear test using the UMT-2 Scratcher to examine the scratch track on the weld surface, which calculates the amount of wear.

The wear tester used is a ball-on-block sliding wear tester. The conditions of the wear test were as follows: counterpart material of φ 10 mm tungsten balls, loading force of 30 N, friction rate of 10 mm/s and duration of 90 min. Wear was computed using the integral method, in which the area of the track on each of the contacted surfaces was measured by means of a (3D) surface profiler. The worn surfaces were analysed using a scanning electron microscope and an energy-dispersive spectrometer. The experimental factors and levels for laser WC/Co/Ni deposits are shown on the left-hand side of Table 2, where eight factors were used based on information from technical manuals and literature. Since many spraying factors have potential effects on the mechanical properties of the deposits. Using trial and error methods, it is not possible to achieve the desired results. Therefore, the design of experiments using statistical methods is required to optimise the process parameters.

## 3. Experimental Design and Method

### 3.1. Experimental Design Based on Orthogonal Array

Robust design, called Taguchi’s method, is a statistical method invented by Genichi Taguchi, which is used in products through optimal design to obtain the best product [35]. It can greatly reduce the variance when it comes to systems with high variables. Orthogonal arrays are a method of designing experiments in which only some of the combinations of factors are usually required. Its array is designed to be balanced against each other, with each factor level being given equal weight. One two-level and seven three-level orthogonal arrays, L18 (2^1^ × 3^7^), were utilised in this study. In total, 18 experiments were to be conducted. Calculations were made for each factor and level based on the arrangement of factors and levels shown in Table 2. Based on the wear-resistant properties, the smaller the wear loss, the better the quality of the experimental product. Therefore, in this case, the signal-to-noise ratio is calculated as follows.

(1) $$S/N=-10Log[\frac{1}{n}(\sum_{i=1}^{n}y_{i}^{2})]$$

The mean values, standard deviations and signal-to-noise (S/N) ratios were calculated as shown in Table 2. This was repeated twice for wear volume loss in each test.

### 3.2. Analysis of Variance

An analysis of variance (ANOVA) has been used to identify the most significant control factors which affect the S/N ratios. An ANOVA was performed using the S/N ratios on the right-hand side in Table 2, and the results are shown in Table 3 with an EDS. It uses the S/N ratios to analyse the effect of control factors on wear volume loss, which can effectively identify important laser cladding parameters that affect the wear volume of the coating. The important control factors associated with the ANOVA were then included in the subsequent analysis, while the optimisation of the parameters was based on the S/N calculations in orthogonal arrays. As shown in Table 4, the effect of each parameter on wear volume can be determined by using analysis of variance (ANOVA). The constituents of the ANOVA table were calculated as sum of squares, degrees of freedom, variance, F-ratio and percentage contribution. A factor effect is statistically significant when it is sufficiently high compared with the experimental error. Therefore, ANOVA can be used to make a more reliable assessment of the relative influence of different factors on the wear volume.

### 3.3. Response Surface Analysis

The response surface methodology (RSM) that was originally developed by Myers and Montgomery is a highly useful statistical tool for improving the quality and productivity of industrial products [36]. It is suitable for designing, developing and manufacturing new products, as well as the improvement and enhancement of current product designs. It also looks for functional relationships in which the response of interest is affected by multiple variables using a regression statistical technique whose goal is to construct an optimised response of a parametric function model. In this study, the wear behaviour of WC/Co/Ni welds during laser cladding involves many controlling factors. Therefore, RSM is used as an alternative method to analyse the wear characteristics. According to an RSM developed by Taguchi, as shown in Table 2 and the following equation, the parameters X_i_ are coded as x_i_:

(2) $$x_{i}=\frac{X_{i+1}-X_{ic}}{\Delta X}$$

where X_i_ is the unsigned value of the ith control factor (i = 1, 2, ..., 4), *X_ic_* is the value of X_i_ at the centroid of the experimental domain, and ΔX is the width of the step. Yet, in this case, the true functional relationship is unknown due to the multivariate system. Therefore, polynomial models are utilised extensively. In this experiment, low-order polynomials related to the experimental domain are used since they are more flexible and simple. The computational matrices of the predicted polynomial model for the functions are

(3) $$Y=\beta_{0}+X^{T}b+X^{T}BX+\varepsilon$$

where,

$$Y=\left[\begin{array}{c} y_{1}\\ y_{2}\\ \vdots \\ y_{n} \end{array}\right],\;b=\left[\begin{array}{c} \beta_{1}\\ \beta_{2}\\ \vdots \\ \beta_{k} \end{array}\right],\;B=\left[\begin{array}{cccc} \beta_{11} & \beta_{12}/2 & \cdots & \beta_{1k}/2\\ \beta_{22} & \cdots & & \beta_{2k}/2\\ & & \vdots & \\ sym & \cdots & & \beta_{kk} \end{array}\right],\;X=\left[\begin{array}{c} x_{1}\\ x_{2}\\ \vdots \\ x_{k} \end{array}\right],\;\varepsilon =\left[\begin{array}{c} \varepsilon_{1}\\ \varepsilon_{2}\\ \vdots \\ \varepsilon_{n} \end{array}\right]$$

The maximal predicted value of **Y** is obtained, and using the least squares method, the coefficients in the regression model can be calculated, and the values of the regression parameters were determined, β. The smallest square estimation of vector, $\hat{B}$, gave a predictive value that is fairly close to the observed value and minimises the sum of squares error. Therefore, the fitting regression model was evaluated to represent the predicted value of *Y_i_*. Therefore, the adjusted regression model, which indicates the predicted *Y_i_* value, was evaluated. A vector of least squares estimates, β, can be obtained by minimising the sum of squared errors. Additionally, the coefficients of regression were determined. It also evaluates the fitted regression model, ${\hat{y}}_{i}$**,** and determines the fit of the model. In this way, the first-order model could be formulated:

(4) $${\overset{ˆ}{y}}_{i}={\overset{ˆ}{\beta }}_{0}+\sum_{i=1}^{k}\;{\overset{ˆ}{\beta }}_{i}x_{i}i=1,2,3,\ldots ,n$$

The second-order model is composed as follows

(5) $${\overset{ˆ}{y}}_{i}={\overset{ˆ}{\beta }}_{0}+\sum_{i=1}^{k}\;{\overset{ˆ}{\beta }}_{i}x_{i}+\sum_{i=1}^{k}\;{\overset{ˆ}{\beta }}_{ii}x_{i}^{2}+\sum \;\sum_{i\leq j}\;{\overset{ˆ}{\beta }}_{ij}x_{i}x_{j}i=1,2,3,\ldots ,n$$

where *x_i_*, *x_j_* and *x_k_* denote the variable levels, *β**_i_*** is the coefficient of the linear equation, *β_ii_* and *β_ij_* are the coefficients of the quadratic equation, ${\hat{y}}_{i}$ are the outputs, and *n* is the numerical number of variables. RSM is a step-by-step process. The steepest path method is used. A set of optimal operating factors and levels are then identified. The predictions of Y are, therefore, remarkably close to the observations. The contour plots in the vicinity of the stationary point in Equations (4) and (5) are also examined in this model.

## 4. Results and Discussion

### 4.1. Microstructure of the Cladding Zone of WC/Co/Ni Welds

**Figure 1:**
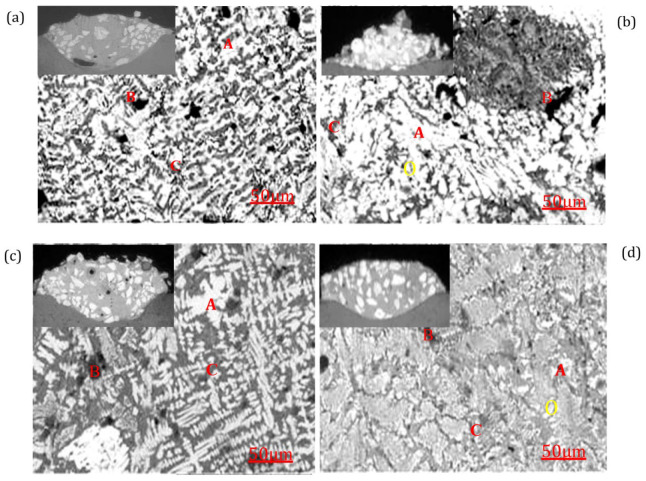
SEM microstructures of various tests with WC/Co/Ni deposits by laser cladding, including (**a**) Test 2 of 100% WC; (**b**) Test 6 of 90% WC-10% Ni; (**c**) Test 12 of 90% WC-10% Co; (**d**) Test 13 of 90% WC-10% Co-10% Ni. Zone A is unmelted carbide, Zone B is porous, and Zone C is melted carbide.

The SEM morphology of the WC weld with added Co/Ni is shown in Figure 1, which shows the etched cross-section of the welds as well as the microstructural evolution of the WC composite. Formation of carbides and precipitates of different structures in the WC/Co/Ni composite welds by laser cladding because of the different contents of the Co/Ni alloys of the WC in the molten pool. Different EDS results for WC mixtures labelled as 100% WC, 90% WC + 10% Ni, 90% WC + 10% Co and 80% WC + 10% Ni + 10% Co in Figure 1 are given in Table 3. As shown in the upper left of Figure 1a–d, the cross-section in the figure shows a highly elevated WC/Ni/Co deposit. It shows melting zones, heat-affected zones and basal areas.

The micrographs in Figure 1 show that some of the dissolved coarse carbide particles are distributed throughout the melt zone. In addition, fine dendritic patterns can be observed in the 100% WC specimen. The coarser carbides are more obvious in the 80% WC/10% Ni/10% Co. The interfacial layer in the melt zone is highly adhesive and flawless. The cladding zone is bright, and the matrix zone is grey. Figure 1a shows the microstructure of the fully melted zone, which contains melted eutectic particles and microcracks in the grey areas. There are irregular needle-like carbide structures in the white areas and larger oxides in some of the black areas. In addition, based on EDS observations, the white area (A) of the melting zone shows W, C and Fe, the black area (B) is enriched in W, O and Fe, with less C and the grey area (C) shows W, C, O and Fe. The reason for this is the different temperature gradient between the white and dark zones. This affects the corresponding structure of the coated deposits. The SEM micrographs of the etched samples are shown in Figure 1a, where the dissolution of WC grains can be seen. The dissolution of WC grains is also confirmed by the EDS shown in Table 3, where a decrease in W and C-based phases and an increase in Fe-based phases with less O are recorded in the melt region. As shown in the upper-left area of Figure 1a for the cladding deposit, the rate of dilution is minor. The surface shows a wavy shape with visible black graphite, large unmelted carbides, pores, cracks and surface irregularities. In addition, the microstructure of unmelted WC particles with 85.167% W elements, several microcracks, randomly distributed dendritic crystalline carbides, a few large particles of oxides and precipitated eutectic granular carbides can be detected in the melt zone of 90% WC-10% Ni (Test 4). The microstructure of the laser-clad weld is shown in Figure 1b, where a large number of white strips of carbide, such as Region A, can be seen, which are presented in Table 3, showing the distribution of the chemical composition of WC/Ni in different regions as black and grey areas obtained by EDS. The bar-shaped carbides are enriched in C, Fe and O but less in Ni. They also contain significant amounts of tungsten. Based on XRD analyses in Ref. [24], these differently shaped precipitated carbides consist of M_6_C, M_12_C and M_7_C_3_. Combining Table 3 and the elements in Region A of Figure 1a,b, it can be seen that the EDX analyses of the laser cladding in Figure 1b show a higher content of the elements W, C and O and a relatively lower content of Fe, compared with Figure 1a.

It can also be seen from the scanning electron microscopy observations that Figure 1a shows a finer distribution of primary precipitated crystalline structures, while Figure 1b shows larger undissolved WC particles, precipitates, coarse dendrites, lumps, grains and graphite. With the above observation, it cannot be avoided that 100% of the WC on the molten layer has been partially dissolved because the W has become less abundant. As shown in the WC/Co welds (Test 13) at the top left of Figure 1c, most of Zone A contains cloudy grey iron clusters and coarse particles of molten WC and is clearly visible at 87.516% W. Coarse WC occurs in the white zone, while the black zone contains 11.148% Fe and 14.295% O with a low of 65.295% W and 3.23% C, and the grey zone has 76.563% W, 4.131% C, 14.402% Fe and a much lower 5.66% O. This indicates that most of the WC composites are concentrated in the white zone. Figure 1b,c shows that the W content of WC/Ni welds is slightly higher than that of WC/Co welds, with an average W content of about 81% W, whereas the WC/Ni welds have more W, C and relatively less O and Fe elements. The influence of the Ni-based alloys on the WC may be greater than that of the Co-based alloys. As shown in the WC/Co/Ni welds in the upper-left area of Figure 1d, the melting zone of Test 14 has a homogeneous distribution of WC particles. No porosity or cracks were found in the cross-sectional images of the structure of the coating. It has lower dilution, favourable metallurgical bonding and lower metallurgical defects. The analysis of Test 12 by EDS is shown in Figure 1d. The white areas show WC (87.291%), oxygen (4.108%), iron (8.700%), Ni (3.717%) and Co (2.906%). The distribution of W in the grey area is similar to that of the white area, with only slight differences in Ni and Co elements. However, the black circular area contains less W (63.965%) and C (3.284%) and more O (24.12%). This indicates a large decrease in WC and an increase in O elements, suggesting the presence of a large amount of oxides in the area.

### 4.2. Wear Behavior of Co/Ni-Mixed WC Welds

**Figure 2:**
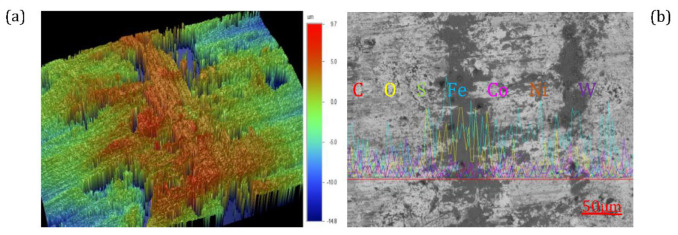

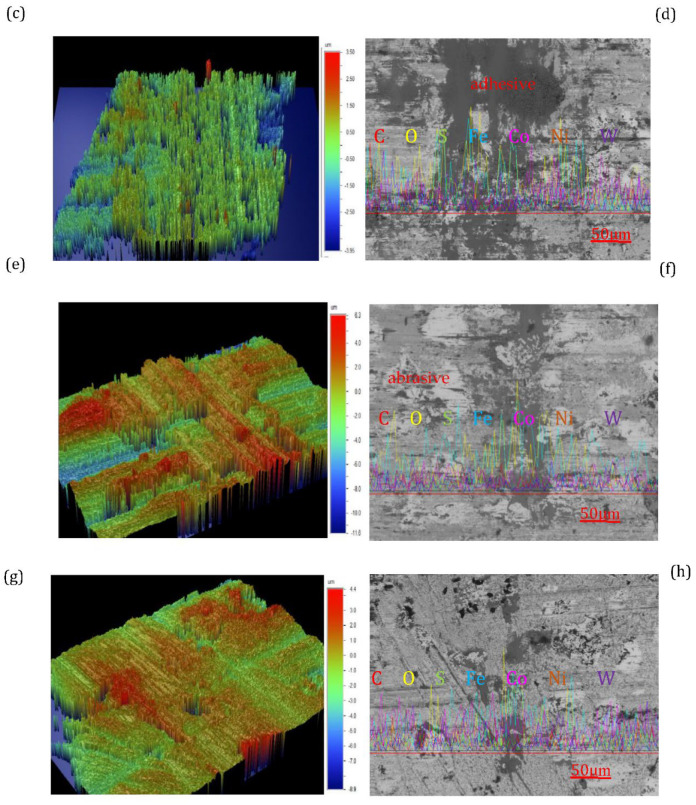
The 3-D topographs of the wear track (**a**–**d**) by wear testing with the cross-sectional profile with WC/Co/Ni deposits by laser cladding, including (**a**,**b**) Test 1 of 100% WC; (**c**,**d**) Test 4 of 90% WC-10% Ni; (**e**,**f**) Test 11 of 90% WC-10% Co; (**g**,**h**) Test 14 of 80% WC-10Co-10Ni.

The results of the wear tests for various WC/Co/Ni coatings are reported in Table 2, and Figure 2 shows the various wear patterns and the distribution of the EDS elements. As shown in Table 2, among the 18 groups of experimental results, we chose 4 groups with different Co/Ni ratios in the WC welds. Tests 1, 4, 11 and 14 had S/N ratios of 59.69, 58.02, 59.42 and 58.22 dB, respectively. The distributions of wear volumes in Test 2, Test 4, Test 13 and Test 14 were 2.47 ± 0.65, 2.00 ± 0.35, 2.36 ± 0.0.36 and 3.05 ± 0.41 × 10^−4^ mm^3^, respectively, which indicate that they are significantly lower than the substrate (28.4 × 10^−4^ mm^3^) by a range of orders of magnitude. A 3D topographic profile of the wear track of Test 2 samples is shown in Figure 2a, along with a 2D profile photograph of the wear containing the elemental distribution of EDS. As shown in Figure 2a, the wear track is not obvious. A few wear wrinkles around the well-defined shallow groove tracks in the red area are clearly visible, along with the occurrence of wear pits. It is possible that the high WC content in the austenitic dendrites has become dispersed and strengthened after wear, thereby improving the wear resistance in this area [4]. The wear pits indicate the presence of micropores and graphite in the WC welds, and the wear mechanism for 100% of the WC can be attributed to the generation of abrasive wear damage. As shown in Figure 2b, the presence of wear cracks is magnified by SEM along with the distribution of EDS elements. Lightly worn surfaces in the grey area are enriched with W, C and small amounts of O, while heavily worn surfaces in the dark area are enriched with Fe and O. As shown in Figure 2c, Test 4, there is a deep furrow in the middle of the wear surface with a depth of about 0.5–1.0 µm, with plough furrows into the green area, and the discontinuous area has less deformation. More wear tracks can be seen in the enlarged photograph in Figure 2c, forming a black area. This may be due to the plastic deformation of the high hard carbide through the mutual wear of the counterpart material of WC. Corresponding to the 3D image in Figure 2c at 90%WC-10%Ni, the abraded areas of hard WC appear to be visible in Figure 2d. This area appears to be the result of oxide formation from the contact of the hard WC with the wear of the counterpart. In addition, the results of the EDS analysis shown in Figure 2d indicate a region of oxide contact on the deep wear surface rich in W, O and Fe, as well as a significant amount of Ni. At 90% WC-10% Co, the 3D morphology of the wear scars on the corresponding wear surfaces in Figure 2e shows the appearance of flat abrasive scars on the wear surfaces with smooth, broad, continuous trajectories, and Figure 2f reflects the wear depth of approximately 2 µm. The mechanism of wear on the wear surfaces described above shows evidence of adhesive wear with severe plastic deformation. This is mainly due to the plastic deformation caused by the precipitation of the initial crystalline phase of the Co binder in the carbide. Similarly, signs of adhesive wear with less plastic deformation also seem to be present, as shown in Figure 2g. The enlarged photo in Figure 2g shows less wear than in Figure 2e. In addition, the EDS analyses shown in Figure 2h also show that the worn surface is enriched in W, O and Fe, but W becomes less enriched. It is clear that the counterpart came into contact with iron debris that was carried into the wear track. The dark-coloured areas have a higher oxygen content, which forms a film of metal oxide during sliding. As observed earlier, increasing the proportion of Co/Ni additives in WC is not effective in improving the wear resistance of laser-clad welds, but it can avoid cracking because welds are toughened. However, adhesive wear is more important than abrasive wear as the percentage of Co/Ni increases. It is evident that the wear resistance of the laser cladding layer is significantly enhanced due to the appropriate amount of Co/Ni in the WC weld.

### 4.3. Effect of Co/Ni Additive Blends on WC Welds

**Figure 3:**
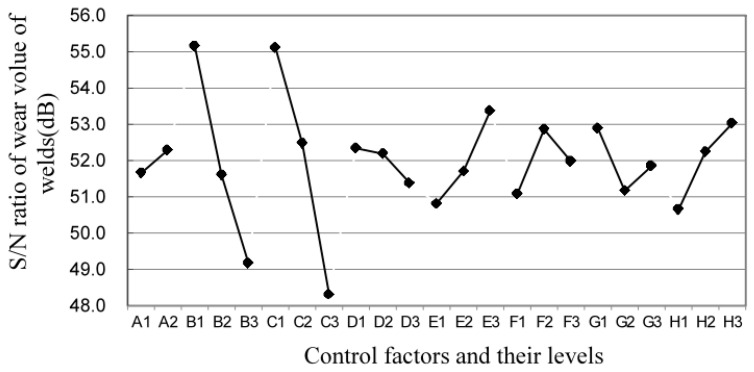
Response plot for S/N ratios of wear volume for WC/Co/Ni welds.

The result of the experiment for L18 is shown in Table 2, where the wear volume losses of each clad weld are calculated from the S/N ratio of each clad weld using the mean and standard deviation. The total wear volume values ranged from 2.1 × 10^−4^ mm^3^ to 8.5 × 10^−4^ mm^3^. The average wear volume of most of the welded specimens is lower than that of the parent material so that the welded joints can be made resistant to wear. Based on the smaller-and-better type, the smaller the volume of wear, the higher the S/N ratio. Therefore, the larger the S/N ratio, the more resistant the coating is to abrasion. The factorial effect table of the experimental results is shown in Figure 3. The effect of the S/N ratio was analysed according to the smaller and better characteristics of the wear volume. The effect of each factor, which is the maximum S/N minus the minimum S/N, is further shown in Figure 3, where factors B, C, E and H have a stronger impact on the variability of the wear volume, while A, D, F and G have a smaller impact. In addition, based on the maximum value of SNR, the best quality is obtained for each factor. Therefore, the maximum value of the S/N ratio for each of the eight factors is A_2_, B_1_, C_1_, D_1_, E_3_, F_2_, G_1_ and H_3_. Furthermore, The S/N ANOVA table was used to confirm the accuracy of the factor analysis table, as shown in Table 4. It consists of the sum of squares, the mean square, the F-test and the contribution ratio. In contrast, the percentage contribution of the eight control factors was greatest for the relative impact of each factor on reducing variation. As shown in Table 4, in this study, factors B, C, E and H were highly significant, while factors A, D, F and G were not significant. This also verifies the validity of the above-mentioned Figure 3. All of these important factors accounted for almost 92.26% of the experimental variation. We confirmed the consistency of the significant factors in the factor-effect analysis. This led us to use them as predictors for the RSM model of the wear volume.

### 4.4. Empirical Model Construction

The design of parameters based on Taguchi’s design and the observed responses are shown in Table 2, which shows the wear volume loss response of the laser-clad weld. Based on the ANOVA in Taguchi’s design, the important parameters were Co% (B), Ni% (C), laser power (E) and scanning height (H) and were included in the analysis of the regression for the wear volume by laser cladding. SPSS22 was used to construct and analyse the experimental data for this design, which makes use of regression modelling. Table 2 lists the designs expressed in natural units, while the coding of the design variables is given by X. The results were calculated for each of the different models. Models containing linear, interactive and quadratic terms were used in response surface methodology (RSM). ANOVA tables containing linear, interaction and quadratic terms created in accordance with Equations (4) and (5). The response surface models with linear, interactive and quadratic terms for the worn volume of the deposits were calculated in the following way:

$$\beta =\left(\begin{array}{l} {\hat{\beta }}_{0}\\ {\hat{\beta }}_{1}\\ {\hat{\beta }}_{2}\\ {\hat{\beta }}_{3}\\ {\hat{\beta }}_{4} \end{array}\right)={\left(X^{T}X\right)}^{-1}X^{T}y=\left(\begin{array}{l} 7.138\\ 0.135\\ 0.165\\ -0.002\\ -0.171 \end{array}\right)\beta =\left(\begin{array}{l} {\hat{\beta }}_{0}\\ {\hat{\beta }}_{1}\\ {\hat{\beta }}_{2}\\ {\hat{\beta }}_{3}\\ {\hat{\beta }}_{4}\\ {\hat{\beta }}_{1}{\hat{\beta }}_{2}\\ {\hat{\beta }}_{1}{\hat{\beta }}_{3}\\ {\hat{\beta }}_{1}{\hat{\beta }}_{4}\\ {\hat{\beta }}_{2}{\hat{\beta }}_{3}\\ {\hat{\beta }}_{2}{\hat{\beta }}_{4}\\ {\hat{\beta }}_{3}{\hat{\beta }}_{4} \end{array}\right)={\left(X^{T}X\right)}^{-1}X^{T}y=\left(\begin{array}{l} 12.339\\ 0.153\\ 0.433\\ -0.005\\ -0.647\\ -0.003\\ 0.000\\ 0.009\\ 0.000\\ -0.001\\ 0.000 \end{array}\right)\beta =\left(\begin{array}{l} {\hat{\beta }}_{0}\\ {\hat{\beta }}_{1}\\ {\hat{\beta }}_{2}\\ {\hat{\beta }}_{3}\\ {\hat{\beta }}_{4}\\ {\hat{\beta }}_{1}^{2}\\ {\hat{\beta }}_{2}^{2}\\ {\hat{\beta }}_{3}^{2}\\ {\hat{\beta }}_{4}^{2}\\ {\hat{\beta }}_{1}{\hat{\beta }}_{2}\\ {\hat{\beta }}_{1}{\hat{\beta }}_{3}\\ {\hat{\beta }}_{1}{\hat{\beta }}_{4}\\ {\hat{\beta }}_{2}{\hat{\beta }}_{3}\\ {\hat{\beta }}_{2}{\hat{\beta }}_{4}\\ {\hat{\beta }}_{3}{\hat{\beta }}_{4} \end{array}\right)={\left(X^{T}X\right)}^{-1}X^{T}y=\left(\begin{array}{l} \;110.527\\ \;10.8901\\ -14.9839\\ \;3.0711\\ -7.5219\\ -0.0941\\ \;0.7052\\ -0.0865\\ \;0.0748\\ -0.2983\\ \;0.0019\\ -0.0564\\ \;0.1169\\ \;0.0848\\ \;0.0016 \end{array}\right)$$

where

$$X=\left(\begin{array}{lllllllllllllll} 1 & 1 & 1 & 1 & 1 & 1 & 1 & 1 & 1 & 1 & 1 & 1 & 1 & 1 & 1\\ 1 & 1 & 0.5 & 0.5 & 0.5 & 0.5 & 0.5 & 0.5 & 0.25 & 0.25 & 0.25 & 1 & 0.25 & 0.25 & 0.25\\ 1 & 1 & 0 & 0 & 0 & 0 & 0 & 0 & 0 & 0 & 0 & 1 & 0 & 0 & 0\\ 1 & 0.5 & 1 & 0.5 & 0 & 0.5 & 0.25 & 0 & 0.5 & 0 & 0 & 0.25 & 1 & 0.25 & 0\\ 1 & 0.5 & 0.5 & 0 & 1 & 0.25 & 0 & 0.5 & 0 & 0.5 & 0 & 0.25 & 0.25 & 0 & 1\\ 1 & 0.5 & 0 & 1 & 0.5 & 0 & 0.5 & 0.25 & 0 & 0 & 0.5 & 0.25 & 0 & 1 & 0.25\\ 1 & 0 & 1 & 1 & 0 & 0 & 0 & 0 & 1 & 0 & 0 & 0 & 1 & 1 & 0\\ 1 & 0 & 0.5 & 0.5 & 1 & 0 & 0 & 0 & 0.25 & 0.5 & 0.5 & 0 & 0.25 & 0.25 & 1\\ 1 & 0 & 0 & 0 & 0.5 & 0 & 0 & 0 & 0 & 0 & 0 & 0 & 0 & 0 & 0.25\\ 1 & 1 & 1 & 0 & 1 & 1 & 0 & 1 & 0 & 1 & 0 & 1 & 1 & 0 & 1\\ 1 & 1 & 0.5 & 1 & 0.5 & 0.5 & 1 & 0.5 & 0.5 & 0.25 & 0.5 & 1 & 0.25 & 1 & 0.25\\ 1 & 1 & 0 & 0.5 & 0 & 0 & 0.5 & 0 & 0 & 0 & 0 & 1 & 0 & 0.25 & 0\\ 1 & 0.5 & 1 & 0 & 0.5 & 0.5 & 0 & 0.25 & 0 & 0.5 & 0 & 0.25 & 1 & 0 & 0.25\\ 1 & 0.5 & 0.5 & 1 & 0 & 0.25 & 0.5 & 0 & 0.5 & 0 & 0 & 0.25 & 0.25 & 1 & 0\\ 1 & 0.5 & 0 & 0.5 & 1 & 0 & 0.25 & 0.5 & 0 & 0 & 0.5 & 0.25 & 0 & 0.25 & 1\\ 1 & 0 & 1 & 0.5 & 0.5 & 0 & 0 & 0 & 0.5 & 0.5 & 0.25 & 0 & 1 & 0.25 & 0.25\\ 1 & 0 & 0.5 & 0 & 0 & 0 & 0 & 0 & 0 & 0 & 0 & 0 & 0.25 & 0 & 0\\ 1 & 0 & 0 & 1 & 1 & 0 & 0 & 0 & 0 & 0 & 1 & 0 & 0 & 1 & 1 \end{array}\right)\;\;\;\;\;\;\;\;\;y=\left(\begin{array}{l} 2.105\\ 2.470\\ 3.570\\ 2.400\\ 3.330\\ 8.835\\ 3.810\\ 7.160\\ 5.105\\ 2.020\\ 2.495\\ 3.330\\ 2.355\\ 3.050\\ 7.365\\ 3.375\\ 4.050\\ 8.485 \end{array}\right)$$

The response surfaces of the wear volume, Y, with respect to the following four important factors, were identified for use in this study and the coefficient estimates (*β*) of the above model were calculated using regression methods. Therefore, the fitted linear, interactive and quadratic terms are shown by Equations (6) and (8).

(6) $$Y=7.1378+0.1348B+0.1652C-0.0019E-0.1713H\;\;\;\;\;\;\;\;\;\;\;\;\;\;Adjust\;R^{2}=0.82$$

(7) $$Y=12.3394+0.1530B+0.4325C-0.0046E-0.647H-0.0034BC-0.0001BE+0.0095BH-0.0002CE\;-0.00005CH+0.000EH\;\;\;\;\;\;\;\;\;\;\;\;\;\;\;\;\;\;\;\;\;\;\;\;\;\;\;\;Adjust\;R^{2}=0.84\;\;\;\;\;\;$$

(8) $$\begin{array}{ll} Y=9.0616\;\;+ & 0.3470B+0.1333C+0.0024E-0.6669H-0.0011BC-0.0002BE+0.0138BH-0.0001CE\\ & +\;\;0.0093CH+0.0004EH-0.0084B2+0.0002C2+0.0000E2-0.0063H2\;\;\;\;\;\;\;\;\;\;\;Adjust\;\;\;\;R^{2}\\ & =0.73 \end{array}$$

Based on Table 2, the experimental data were fitted using the above model. The equations for fitting the RSM model use Equations (3) and (5) to determine Equations (6) and (8) for wear volume loss. The results of ANOVA are shown in Table 5, where the value of F was calculated as smaller than 0.05, meaning “Prob > F”, indicating a remarkable and robust regression model. The outcome of the ANOVA for the linear and interaction models revealed that both of these models were found to be remarkably important with ‘Prob > F’ values of 0.000 and 0.0032, respectively, whereas the quadratic term was not significant with ‘Prob > F’ value of 0.127. In addition, the adjusted R^2^ values for the coefficients of determination of regression for the linear, interaction and quadratic models were 0.82, 0.84 and 0.73, respectively. However, the higher values of the adjusted coefficient of determination for the interaction model suggest that these models have higher significance. On the other hand, the significant terms of the interaction model, when compared to the linear and quadratic models, were similarly identified, as shown in Table 6. Therefore, the interaction model was picked for prediction.

### 4.5. Empirical Model Analysis

**Figure 4:**
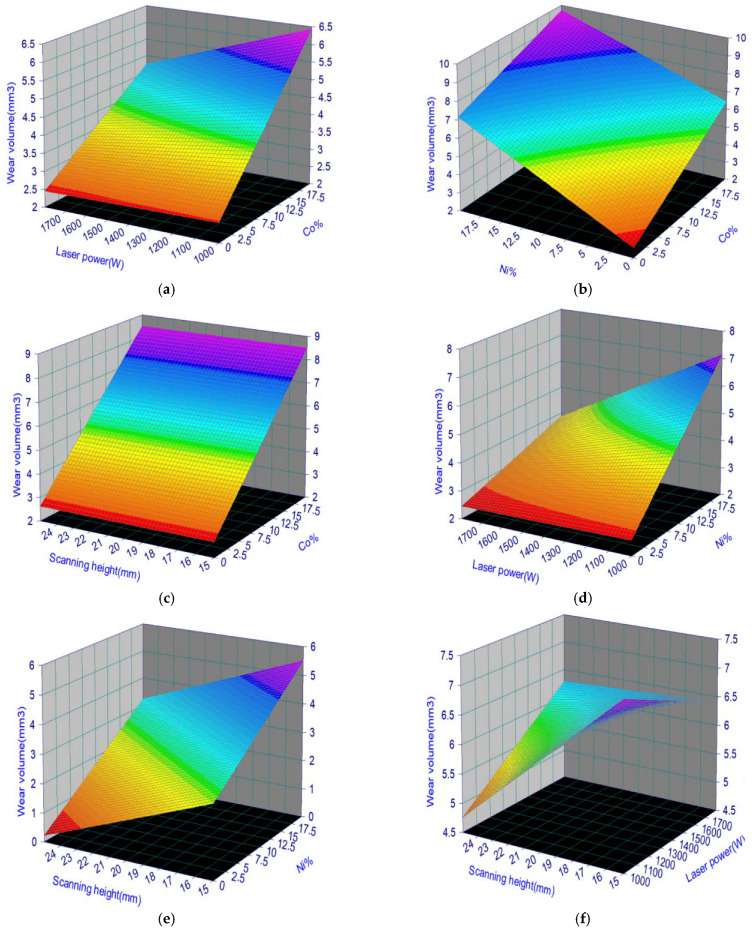
Contour plots of wear volume (×10^−4^) for the interaction model. (**a**) The effect of Co% and laser power on wear volume losses. (**b**) Saddles containing Co% and Ni%% were used in the WC results. (**c**) Surface plane plot of wear volume in Co% content vs. scanning height. (**d**) Saddle contour plots in Ni contents and laser power on wear volume. (**e**) Planar pattern with scanning height and Ni%. (**f**) Saddle plot of laser power and scanning height on wear volume.

**Figure 5:**
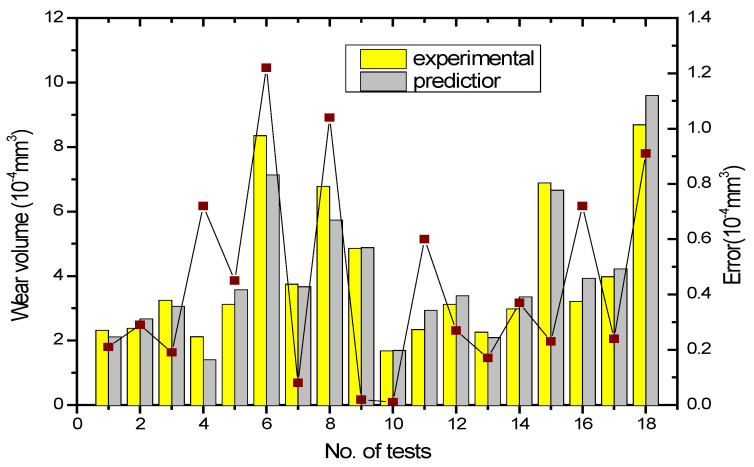
Comparison of the histograms and errors of the predictions with the experimental data obtained by means of an interaction model.

For a better understanding of the properties of WC/Co/Ni coatings, we introduce the contours of the interaction functions used by Equations (6) and (8). The interaction function is shown in Figure 4, which displays a three-dimensional contour plot of the response surface for the wear volume yield under significant control factors. The contour plots of the interaction model are shown in Figure 4, where two of the variables are held at the centre level, and the other two variables vary within the experimental range. The contour shape of the 3D map is nearly planar with a saddle-type contour. In Figure 4, the shape of the saddle pattern indicates a significant interaction between the variables, whereas the shape of the planar profile does not. The rough plan view in Figure 4a shows the effect of Co% and laser power on wear volume losses. The results show that the increase in Co% has great effects on the increase in wear loss when the laser power is in the range of 1000–1800 W. This indicates that too much Co% in the WC results in unfavourable wear resistance of the cladding zone. The saddle profile in Figure 4b shows that a lower Co% content with less Ni% used in WC results in a lower wear volume of the coating. The saddle contours already showed that the amount of wear on the coatings was limited by the interaction of Co% and Ni% content. Therefore, the amount of both needs to be controlled appropriately. In Figure 4c, the pattern is similar to that of Figure 4a, where the wear volume is smaller when the Co% content is lower and the scanning height is between 15 mm and 25 mm. As shown above, the effect of Co% content on the amount of wear is much greater than that of the scanning height. As shown in the saddle contour plot in Figure 4d, the effect of laser power on wearing volume is not significant at lower Ni% amounts. When the amount of Ni% is gradually increased, the two effects are more reciprocal, so the wear volume increases significantly. In any case, the effect of Ni% on the wear volume is much higher than that of laser power when the laser power is increased from 1000 W to 1800 W. Figure 4e shows the 3D photographs and contour plots of the saddle pattern when varying the scanning height and Ni% content. The wear volume of the WC weld is minimised when the scanning height reaches 25 mm, and the Ni% content is close to zero. As shown in Figure 4e, the Ni% content has a greater influence on the wear volume of WC welds than the scanning height. Figure 4f shows the relationship between the laser power and scanning height on the wear volume, where a better wear volume is obtained when the scanning height is close to 25 mm, and the laser power is fixed at 1000 W. Compared with the other plots mentioned above, it is relatively high, and it is unable to achieve the desired results. However, the effect of Ni% and Co% on the wear volume is greater than that of scanning height and laser power, which is in good agreement with the results in Table 6. Contour maps are used to find the best areas in the 3D images, and they clearly show the relationship between the amount of wear and key factors. In addition, it provides useful information about the anti-wear properties of the coating. To verify the predictive ability of the interaction model, the experimental and predicted values were compared. Figure 5 shows the histograms of the experiments and predictions and the results of the errors in the predictions of the interaction model. Comparisons were made across all experiments, and the results showed that the predictions were more reliable in most cases, except for experiments 6, 8 and 18, where the errors were more than 10%. However, the distribution of the prediction errors of the interaction model fluctuates smoothly, and only three of them had errors of prediction that exceeded the standard deviation of the experimental values by more than two times. This means that the predicted values are remarkably close to the experimental values, indicating that this model is trustworthy. Therefore, it can be concluded that the developed interaction model is effective in predicting the wear volume characteristics of laser-coated WC/Co/Ni welds.

### 4.6. Confirmation Experiments

**Figure 6:**
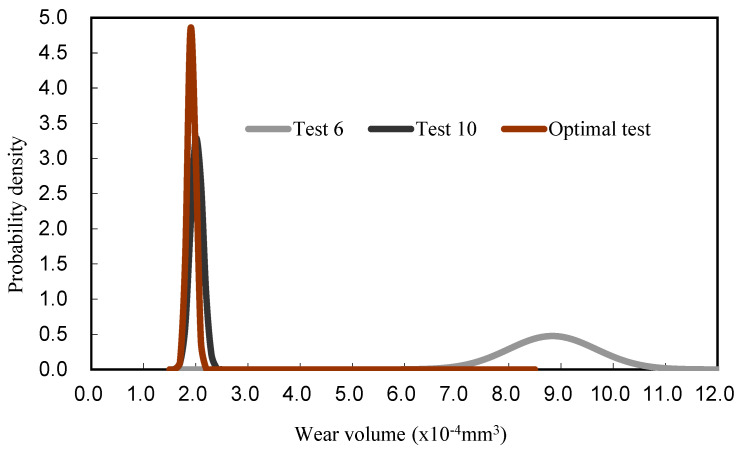
Confirmation of the optimum test based on the orthogonal table for the amount of wear of the coated surfaces and comparison of the selected Test 6 and Test 10 with Gaussian function.

To verify the validity of the performance predictions of the best control parameters and gain a better understanding of the influence of the parameters on the wear behaviour of the coatings, the optimum value of each factor in the orthogonal table is shown in Figure 3, which was calculated based on the maximal S/N value of the level of each factor. The optimal setting for the factorial levels is A_2_B_1_C_1_D_1_E_3_F_2_G_1_H_3_. The worst test (6) was chosen with the colour black, and the better test (10) with the colour grey in all 18 sets of orthogonal arrays. These two tests were compared with the best test using brown. In Figure 6, the probability density of wear volume is displayed, which compares the best test with Test 6 and Test 10. The less wear you have, the closer you are to the left side of the graph. The thinnest solid curve on the left side of Figure 6 indicates a small standard deviation from the minimum wear in the best test. It is clear that the optimum setting of the control factor has very little effect on the variation, which indicates good quality characteristics. Table 2 shows that WC/Co/Ni welds have better anti-wear properties under optimum conditions, which indicates that wear is significantly improved in the substrates by laser cladding. As a whole, the optimal setting of the control factors has been shown to be sufficiently robust against the effects of noise, thereby achieving better reproducibility.

## 5. Concluding Remarks

In this study, laser cladding of WC/Co/Ni welds was used. The mechanical properties of the coating were studied. The mechanical properties of the coatings were improved by optimising the welding and developing RSM models using statistical tools. The following conclusions were reached:

(1) The microstructure of the white area of the melting zone is dominated by dendritic carbides, which are close to 81% or more W, while the percentage of C is about 3–5%. In addition, EDS analyses of the wear areas showed that the areas of resistance to wear contain areas of carbides that are high in W and O content but low in Fe content.
(2) The wear resistance of the WC/Co/Ni welds varied considerably, and that of WC with Co and Ni was much higher than that of WC/Co or W/Ni. Compared with the substrate, the wear resistance of the coatings in this study increased by about five times or more, indicating that the coatings have good wear resistance.
(3) The resulting analysis of variance showed that the effect of four variables on the wear volume was obvious, in which the factors Co%, Ni%, laser power and scanning height were significant in accounting for more than 92.26% of the total variance.
(4) Linear function, interaction function and second-order functions were used in RSM, whose Adjust-R^2^ coefficients were 0.82, 0.84 and 0.73, respectively. The interaction model demonstrated good predictive power in the experimental area.
(5) WC welds with Co/Ni additions by laser cladding are effective against substrate wear, thereby verifying that the interaction model provides a more reliable model for the cladding process.

## Figures and Tables

**Table 1:** Chemical composition of WC, Co-based and Ni-based alloys used in WC/Co/Ni welds.

Symbol	Weight of Element (wt%)						Particle Size (μm)
WC	W	C	Fe	Co	Cu	S	60–100
	98.3	1.3	0.0013	0.0005	0.0013	0.0015	
Co	Co	Cu	Fe	Al	C	S	40–150
	99.9	0.05	0.065	0.05	0.02	0.009	
Ni	Ni	Co	Fe	Al	C	S	40–150
	99.9	0.0013	0.0013	0.01	0.01	0.0015	

**Table 2:** The 18 tests of wear volume with S/N ratios for control parameters and levels by laser cladding.

No. of Tests	Substrate	Co%	Ni%	Preheat Temperature	Laser Power	Carrier Flowrate	Scanning Speed	Scanning Height	Wear Volume		S/N Ratio
	A	B	C	D	E	F	G	H	10^−4^ mm^3^		dB
1	1	0	0	25	1000	1400	2	15	2.11	0.23	60.61
2	1	0	10	100	1400	1600	4	20	2.47	0.65	59.2
3	1	0	20	200	1800	1800	6	25	3.57	0.75	57.84
4	1	10	0	25	1400	1600	6	25	2.40	0.35	59.69
5	1	10	10	100	1800	1800	2	15	3.33	0.52	58.02
6	1	10	20	200	1000	1400	4	20	8.84	0.84	56.52
7	1	20	0	100	1000	1800	4	25	3.81	0.49	57.23
8	1	20	10	200	1400	1400	6	15	7.16	0.95	56.81
9	1	20	20	25	1800	1600	2	20	5.11	0.87	56.1
10	2	0	0	200	1800	1600	4	15	2.02	0.12	60.7
11	2	0	10	25	1000	1800	6	20	2.50	0.35	59.27
12	2	0	20	100	1400	1400	2	25	3.33	0.62	58.02
13	2	10	0	100	1800	1400	6	20	2.36	0.36	59.42
14	2	10	10	200	1000	1600	2	25	3.05	0.41	58.22
15	2	10	20	25	1400	1800	4	15	7.37	0.75	56.7
16	2	20	0	200	1400	1800	2	20	3.38	0.42	57.9
17	2	20	10	25	1800	1400	4	25	4.05	0.51	56.97
18	2	20	20	100	1000	1600	6	15	8.49	0.49	56.25

**Table 3:** The weight percent chemical composition of the atomic concentration of WC containing Co/Ni additives by EDS.

No. of Tests		Atomic Concentration (%)					
		W	C	O	Fe	Ni	Co
WC	A	85.1670	4.7740	0.8860	9.1930	0.0000	0.0000
	B	68.1220	2.7170	7.3380	21.7420	0.0000	0.0000
	C	83.0990	5.2300	2.4120	3.2130	0.0000	0.0000
WC/Ni	A	86.2090	4.0230	5.8840	2.6240	0.2600	0.0000
	B	72.6080	4.8970	8.2480	12.6240	0.7810	0.0000
	C	83.0870	5.0120	2.4120	4.6030	4.4900	0.0000
WC/Co	A	87.5160	4.4640	2.3100	4.6520	0.0000	0.7250
	B	65.2950	3.2300	14.2950	11.1480	0.0000	0.0920
	C	76.5630	4.1310	5.6600	12.4020	0.0000	0.0810
WC/Co/Ni	A	87.2910	3.6670	4.1080	8.7000	3.7170	2.9060
	B	63.9650	3.2840	24.2120	5.5740	1.6560	1.2300
	C	82.0270	4.5670	5.2750	4.7990	1.9350	1.4270

**Table 4:** Analysis of variance.

Symbol	Sum of Squares	Degree of Freedom	Mean Square	F-Test	Contribution Percent
A	1.82	1	1.82	7.63	0.59
B	108.36	2	54.18	226.6	34.82
C	140.9	2	70.45	294.64	45.28
D	3.17	2	1.58	6.64	1.02
E	20.24	2	10.12	42.33	6.51
F	9.56	2	4.78	20	3.07
G	9.033	2	4.51	18.88	2.9
H	17.59	2	8.79	36.78	5.65
Error	0.47	2	0.23	1	0.15
Total	311.18	17			100

**Table 5:** Analysis of variance of response of wear volume based on linear, interaction and quadratic functions.

Symbol	Degree of Freedom	Sum of Squares	Mean Square	F-Test	Prob > F	Adjust-R^2^
First-order model	4	70.54	17.63	20.49	0.0000	0.82
Interaction model	10	76.23	7.62	9.71	0.0032	0.84
Second-order model	14	77.86	5.56	4.31	0.1273	0.73

**Table 6:** The estimated coefficient of regression and *p*-value for linear, interaction and quadratic models.

Source	Second-Order Model			Source	Interaction Model		
	Coefficient	t-	Prob > F		Coefficient	t-	Prob > F
	Estimate	Statistic			Estimate	Statistic	
Intercept	9.0616	0.5790	0.6032	Intercept	12.3394	2.1465	0.0690
*B*	0.3470	0.9442	0.4147	*B*	0.1530	0.8176	0.4405
*C*	0.1333	0.3310	0.7624	*C*	0.4325	1.9486	0.0924
*E*	0.0024	0.1687	0.8768	*E*	−0.0046	−1.3315	0.2248
*H*	−0.6669	−0.5984	0.5918	*H*	−0.6474	−2.0972	0.0742
*BC*	−0.0011	−0.1593	0.8835	*BC*	−0.0034	−0.7425	0.4819
*BE*	−0.0002	−1.0098	0.3870	*BE*	−0.0001	−1.3688	0.2134
*BH*	0.0138	0.9863	0.3967	*BH*	0.0095	1.0201	0.3416
*CE*	−0.0001	−0.6562	0.5585	*CE*	−0.0002	−1.6971	0.1335
*CH*	0.0093	0.6078	0.5862	*CH*	−0.0005	−0.0593	0.9544
*EH*	0.0004	1.3075	0.2822	*EH*	0.0003	1.5289	0.1701
*B*2	−0.0084	−0.9502	0.4121	Source	First-order model		
*C*2	0.0002	0.0236	0.9827		Coefficient	t-	Prob > F
*E*2	0.0000	−0.6321	0.5722		Estimate	statistic	
*H*2	−0.0063	−0.2081	0.8485	Intercept	7.1378	4.7933	0.0004
				*B*	0.1348	5.0347	0.0002
				*C*	0.1652	6.1673	0.0000
				*E*	−0.0019	−2.8876	0.0127
				*H*	−0.1713	−3.1988	0.0070

## Data Availability

The raw data supporting the conclusions of this article will be made available by the authors on request.
